# Synchronous malignant renal mass in patient with a Lung cancer: case report and literature review

**DOI:** 10.11604/pamj.2015.20.22.5541

**Published:** 2015-01-07

**Authors:** Aicha Mazouz, Lamiae Amaadour, Ihsane Souaf, Hinde El Fatemi, Afaf Amarti, Mohamed Ait Erraisse, Essaadia Oubelkacem, Touria Bouhafa, Yassir Tahiri, Mohammed Fadl Tazi, Soufiane Mellas, Samia Arifi, Nawfel Mellas

**Affiliations:** 1 Department of Medical Oncology, Hassan II University Hospital, Fez, Morocco; 2 Department of Pathology, Hassan II University Hospital, Fez, Morocco; 3 Department of Radiotherapy, Hassan II University Hospital, Fez, Morocco; 4 Department of Urology, Hassan II University Hospital, Fez, Morocco

**Keywords:** Synchronous malignant renal mass, non-small cell lung cancer, synchronous primary tumor, differentiating features

## Abstract

The finding on imaging (computed tomography scan or magnetic resonance imaging) of synchronous malignant renal mass in patient with an active nonrenal malignancy without renal specific symptoms is not frequent and diagnostic evaluation can be challenging. We describe a 54-year-old Moroccan male former chronic smoker who presented to our hospital with dry cough and impairment of the performance status. The imaging found a tumor mass in the left upper lobe of the lung associated to mediastinal lymph node and a scanno-guided biopsy of this tumor showed a non small cell lung cancer. The radiological staging revealed a solitary renal mass in the right kidney. The patient received firstly two cycles of a lung cancer chemotherapy with a partial response in the lung and a stability of the renal mass. Consequently, he underwent a scanno-guided biopsy of this mass which confirmed a synchronous clear cell renal carcinoma. The patient got chemo radiotherapy for the lung cancer and then after that he got a partial nephrectomy. He is still under a good control with more than 2 years after the initial diagnosis.

## Introduction

The finding on imaging (computed tomography scan or magnetic resonance imaging) of synchronous malignant renal mass in patient with an active nonrenal malignancy without renal specific symptoms is not frequent [1] and diagnostic evaluation can be challenging because renal tumor may be either a synchronous primary renal tumor or a metastatic disease. However, the kidney is a rare site of metastatic disease from primary tumors [2]. The same developing two or multiple different synchronous primary tumors in the same patient is very uncommon in the kidney and the lung [3]. Here we describe a case report with synchronous primary tumors of the non-small cell lung cancer (NSCLC) and the clear cell renal carcinoma (CCR), and discuss through it the problem of differentiation between synchronous primary renal neoplasm and metastasis to the kidney from an active nonrenal malignancy like a NSCLC, when we find a synchronous malignant renal mass because it is crucial for further management and prognosis [4].

## Patient and observation

**Figure 1:**
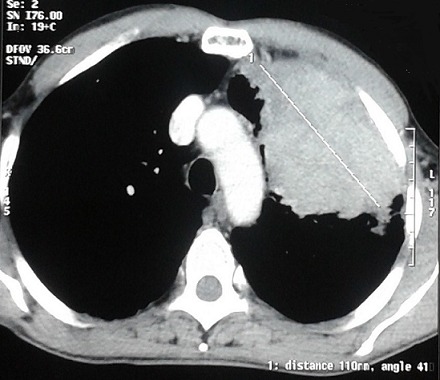
CT scan of the chest showing a tumor mass in the left upper lobe which measures 90 × 76mm

**Figure 2:**
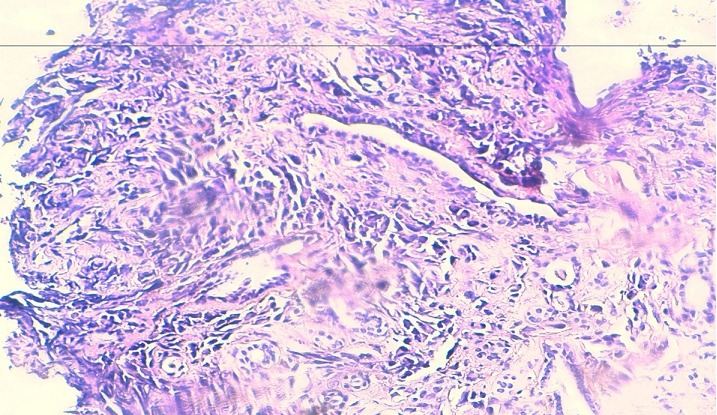
A biopsy of the tumor mass of the lung

**Figure 3:**
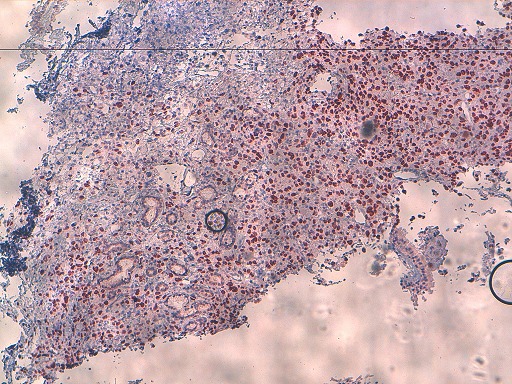
A biopsy of the tumor mass of the lung. Immunohistochemical staining of this biopsy showed a diffusely expression of the TTF1

**Figure 4:**
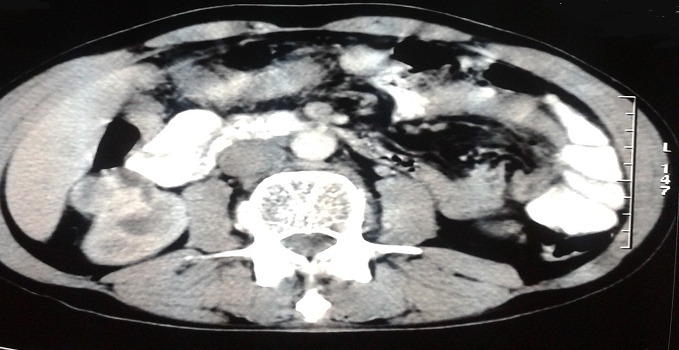
CT scan of the abdomen showing a solid tumor mass in the right kidney measuring 34mm with exophytic development and moderate enhancement

**Figure 5:**
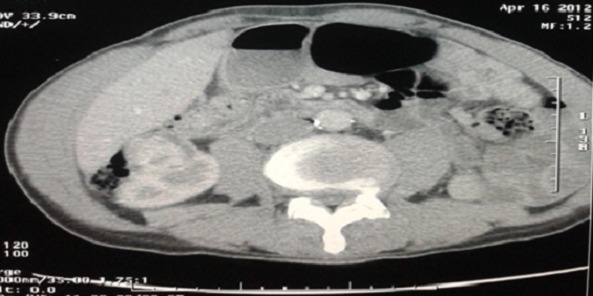
Evaluation after 2 cycles of chemotherapy with a CT scan of the abdomen. CT scan cut showing a stability of the renal mass according to RECIST 1.1

**Figure 6:**
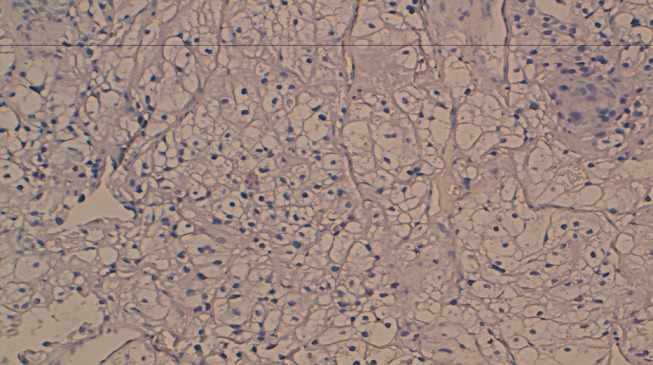
A biopsy of the renal tumor. Histologic sections (HES×10) of biopsy of the renal tumor showed a proliferation of tumor cells with clear cytoplasm

**Figure 7:**
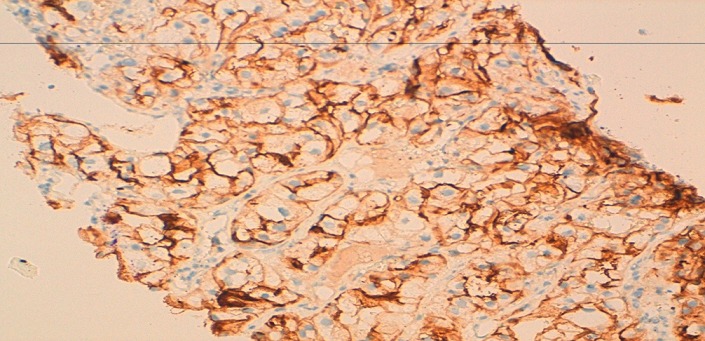
A biopsy of the renal tumor. Immunohistochemical staining of this biopsy showed a diffusely expression of the CD10

Written informed consents were obtained from the patient for publication of this case report and any accompanying images. A 54-year-old Moroccan male former chronic smoker, presented to our hospital with dry cough, left chest pain and impairment of the performance status (PS) in the last six months. The physical examination was normal and the PS was scored 1. A standard chest radiograph showed a left upper lobe opacity very suspected and a computed tomography (CT)scan of the chest confirmed this tumor mass in the left upper lobe which measures 90×76mm (Figure 1) associated to a lymph node of the anterior mediastinal chain. The patient underwent a scanno-guided biopsy of the lung tumor which showed a localization of moderately differentiated adenocarcinoma (Figure 2). On immunohistochemical staining, the thyroid transcription factor-1(TTF1) and the cytokeratin 7(CK7) were diffusely positive (Figure 3) and the CK20 was negative confirmed a NSCLC. In order to classify this tumor, the patient got a CT scan of the brain, the abdomen and the bone which revealed only a solitary renal mass in the lower pole of the right kidney which was solid, small measuring 34mm with exophytic development and moderate enhancement (Figure 4). The NSCLC was classified according to the Union for International Cancer Control (UICC) system (7th edition) stage IV with metastasis to the kidney or inoperable stage III with a probable synchronous primary renal neoplasm. The patient received NSCLC chemotherapy with cisplatin (80mg/m^2^, day 1) and vinorelbin (25mg/m^2^, day 1 and 8). At evaluation after 2 cycles we observed a partial response in the lung with a stability of the renal mass (Figure 5) according to RECIST1.1. The diagnosis of metastasis to the Kidney was feared and the patient got a scanno-guided biopsy of this renal mass that showed a proliferation of tumor cells with clear cytoplasm (Figure 6) which expressed the cluster of differentiation 10 (CD10) and the epithelial membrane antigen (EMA) (Figure 7) but did not express the vimentin and the CK7, confirming a synchronous CCR which was classed stage I. The patient got chemo radiotherapy for the lung cancer using photon 18mV with 70 Gy (2Gy/day) concomittent with cisplatin and vinorelbin with radiologic response and then after that he got a partial nephrectomy. The patient is still under a good control with more than 2 years after the initial diagnosis.

## Discussion

In patient with a NSCLC, we can find on CT a synchronous malignant renal mass that suggests metastasis to the kidney but it can also have another origin like a synchronous primary renal neoplasia. However, the kidney is a rare site of metastatic disease from primary tumors. Abrams et al. reported in autopsy study of 1000 autopsied cases who died of disseminated carcinoma that 12.6% had metastasis to the kidney which was the tenth most common target of metastatic spread and the tumors reported to more frequently metastasize to the kidney are lymphoma, lung and melanoma [2]. Also, synchronous primary tumors involving the kidney and a lung cancer are uncommon [3]. However, it is important to differentiate between synchronous primary renal neoplasm and metastasis to the kidney with different treatments and prognosis [4].

To compare the incidence between the metastasis to the kidney and primary renal neoplasia, Patel U et al [5] published the results of a retrospective study of 2340 patients with an active primary nonrenal malignancy who presents a renal mass at the diagnosis or at follow-up. In this study, the lung cancer was the third reported active primary nonrenal malignancy. The results of the contemporaneous frequency of metastases within the kidney as opposed to primary renal tumors revealed respectively 0.9% versus 0.6%on review of the histologic records (biopsy or surgical extirpation) and follow-up studies. These rates are close which requires knowing the differentiating between these two entities with different treatments and prognosis. The authors concluded that the imaging (CT) may have a diagnostic approach by analyzing the consistency of the renal mass and its location. As a result, the metastasis to the kidney was significantly a solid and endophytic mass, and not significantly localized mainly in the upper pole of the kidney. However, previous studies [6] have observed two common features which were not significantly different in this study; it was a lack of or poor enhancement in kidney metastasis and bilaterality.

For the positron emission tomography scan with fludeoxyglucose (FDG), it can be do in the case of a solitary renal mass wherein it can detect a metastasis to the kidney if the primary tumor is not FGD-avid. However it is not very highly performant to the detection of a primary renal neoplasm with low sensitivity [7].

For the biopsy, we do not have guidelines for performing a renal mass biopsy in patients with a history of a nonrenal malignancy. Ricardo F et al [8] published the results of a retrospective study of 100 patients with nonrenal malignancies diagnosed with renal mass at presentation or follow-up in order to evaluate their experience with this dilemma and to formulate management guidelines. The renal mass histology was available for all patients after nephrectomy or biopsy and the lung cancer was noted in 13% of the nonrenal malignancies. The authors concluded that in case of a metastatic nonrenal malignancy, the indications to perform a biopsy for a renal mass should be based on the prognosis of the patient and the importance of differentiating the presence of an additional metastatic organ site versus another primary malignancy. For example, the patients with a nonrenal primary tumor with a long life expectancy and a solitary small renal mass are more likely to benefit from surgical extirpation of their renal tumor rather than a biopsy. Small renal mass in poor prognosis primary may benefit from surveillance.

However, if doing a biopsy of the renal mass and the histology alone is not conclusive, immunohistochemistry is useful because TTF-1 which is a typical marker for adenocarcinoma in the lung is negative in primary renal neoplasms [9] and a CCR which represents 80% of the kidney cancer expressed often the CD10 and the vimentin with no expression of the CK7.

Once the diagnosis is confirmed, definitive therapy should follow. In case of the primary renal neoplasm, surgery is the gold standard [10]. For the metastatic disease, the therapy will depend on the primitive tumor histology.

Our patient had an inoperable locally advanced NSCLC with a renal mass which was asymptomatic, small and solid but it was localized in the lower pole of the kidney with exophytic development and moderate enhancement. We did not have enough criteria for distinguishing between a metastasis to the kidney and a primary renal neoplasia. We decided to start an induction NSCLC chemotherapy which has a poor prognosis with 2 cycles and at evaluation we observed a stability of the renal mass that suggestive its primitive origin. The patient underwent a biopsy and not a surgical extirpation of the renal mass to confirm a synchronous CCR. Then after that, he got a chemo radiotherapy followed by a partial nephrectomy which is the gold standard of the primary renal neoplasm in this case.

## Conclusion

In patients with an active nonrenal malignancy like the NSCLC, if the imaging shows a solitary malignant renal mass, the differentiation between a metastatic mass and a primary renal neoplasia should be done. Metastasis is more likely asymptomatic, small and solid mass with endophytic development and poor enhancement. The indication of a renal biopsy or surgical extirpation should be discussed depending on the prognosis of the patient and the importance of differentiating between these two entities with different treatments and prognosis.
